# Reducing recalcitrance of black pepper to *Agrobacterium*-mediated transformation: an efficient way through nucellar apomixis to establish transgenic and genome-edited plants at high frequency and scale-up through bioreactor

**DOI:** 10.1093/hr/uhag067

**Published:** 2026-02-28

**Authors:** Shina Sasi, Saranya Krishnan, Martin Kottackal, Khaled MA Amiri

**Affiliations:** Khalifa Center for Genetic Engineering and Biotechnology, PO Box 15551, Al Ain, United Arab Emirates; Khalifa Center for Genetic Engineering and Biotechnology, PO Box 15551, Al Ain, United Arab Emirates; Khalifa Center for Genetic Engineering and Biotechnology, PO Box 15551, Al Ain, United Arab Emirates; Khalifa Center for Genetic Engineering and Biotechnology, PO Box 15551, Al Ain, United Arab Emirates; Department of Biology, College of Science, United Arab Emirates University, PO Box 15551, Al Ain, United Arab Emirates

## Abstract

Nucellar apomixis is truly clonal and is a powerful tool for broadening the genetic base of crops. Black pepper (*Piper nigrum* L.), the ‘King of Spices’ is difficult to improve through conventional breeding. Although transgenesis and genome editing are prime strategies for rapid crop improvement, recalcitrance hinders genetic modifications. Here, we report a highly efficient *Agrobacterium*-mediated procedure for generating genetically modified black pepper plants using nucellar apomixis-derived embryos of the varieties Sreekara and Karimunda, with a 99% survival rate. Both *Agrobacterium tumefaciens* and *Agrobacterium rhizogenes* were efficient in transformation, and the AGL1 strain harboring the plasmid with *mgfp* achieved >90% frequency following 20 min in the infection medium, 30 s sonication, 10 min vacuum infiltration, and 4 days of cocultivation. Sugar type determined embryonal taproot development and soil establishment. Glucose-supplemented medium produced plantlets with well-developed root systems that displayed a high expression of *PnPIN2.* Transgenic plantlets survival *ex vitro* from glucose-supplemented liquid medium was 99%. The genome-editing efficiency of *Pds* using CRISPR/*Cas9* was 89%. Agroinfiltration of black pepper in this study is useful for high-throughput screening of disease resistance. Composite plants of black pepper generated at >60% efficacy is an easy strategy to develop plants expressing disease-resistant genes in roots to reduce yield loss, especially by root-rot. This study demonstrates that black pepper is an easy-to-transform crop, which reinforces speedy trait development through genetic modifications. Scale-up using temporary immersion bioreactors in this study fast-track high throughput accomplishment of untransformed/transformed/genome edited plants empower the market demand for black pepper.

## Introduction

Black pepper (*Piper nigrum* L., 2*n* = 52; Piperacae) is celebrated as the ‘King of Spices.’ It is a perennial woody vine that grows on supporting trees, poles, and trellises. It is native to the Malabar Coast of India and has been used in culinary applications since at least 2000 BCE. It is now cultivated in most tropical and subtropical regions, with production primarily in Vietnam, Indonesia, Brazil, India, Sri Lanka, China, Malaysia, and Cambodia. Black peppercorns are the most widely traded spices in the world (traded as collateral currency during the Middle Ages). The total global production of black pepper, also known as black gold, is around 3 50 000–4 00 000 tonnes per annum, and the global market size reached US$ 4.5 Bn in 2024 and is projected to reach US$ 7.99 billion by 2032, with a growing CAGR of 4.5% from 2022 to 2032 (https://www.imarcgroup.com/spices-seasonings-market). The use of black pepper in the pharmaceutical and nutraceutical fields is due to its antioxidant and antibacterial properties, and its use in skin-care products increases the demand every year. The global black pepper oleoresin market in 2024 is valued at over US$ 55 Bn and is projected to cross US$ 92.63 Bn by 2032 at a CAGR of 7.73% from 2024 to 2031 (https://www.transparencymarketresearch.com/black-pepper-oleoresin-market.html)*.*

The black pepper market is experiencing year-on-year growth. However, the supply does not meet market demand, mainly because of the intensive crop losses in black pepper cultivating countries worldwide, especially in Vietnam, India, and Brazil. Climate change and diseases caused by fungi, bacteria, viruses, nematodes, and insect pests are the main setbacks in the yield of black pepper plants. Global pepper production is estimated to decrease by 3% annually. Of the productivity loss, the foot root/quick wilt disease caused by *Phytophthora capsici* stands first, followed by other diseases such as phyllody, black berry, stunt disease, slow decline, anthracnose, and infection of root-knot ‘nematode’ (*Meloidogyne incognita*), *Piper* yellow mottle virus, and insect pests like pollu beetle (*Lanka ramakrishnai*) and top shoot borer (*Cydia hemidoxa*) (https://en.vikaspedia.in/viewcontent/agriculture/crop-production/integrated-pest-managment/ipm-for-spice-crops/ipm-strategies-for-black-pepper/black-pepper-diseases-and-symptoms).

Black pepper improvement aims to achieve a high crop yield by improving disease and pest resistance, tolerance to resilient climate change (multiple abiotic stresses), oleoresin content, other important secondary metabolites, and flavor. The ICAR-Indian Institute of Spices Research (IISR) at Kozhikode, Kerala, India, owns the world’s most extensive collection of black pepper germplasm [1], which consists of 3467 accessions comprising exotic and wild relatives (http://www.spices.res.in/pages/black-pepper). The variety Sreekara, obtained by selection from Karimunda (KS-14), the ancestral pepper, was released in 1990 by the ICAR-IISR, India. As the breeding cycle requires 10 to 20 years, the conventional breeding of black pepper takes several years. However, conventional breeding has produced high-yielding varieties that are still prone to biotic and abiotic stresses.

Nucellar embryony, widely reported in citrus varieties, is a type of apomixis in which nucellar embryos originate from the nucellus tissue of the ovule, independent of meiosis and sexual reproduction [2]. As nucellar apomictic progenies fix the traits of the mother plants, they are considered as a potential tool in plant breeding. High-frequency nucellar somatic embryogenesis *in vitro* fast-tracks high-throughput genetic manipulation, including genome editing. Nucellar somatic embryogenesis has been reported in black pepper [3, 4], but has not been explored for genetic manipulation, especially genome editing, because of its recalcitrance. The generation of genetically modified plants by *Agrobacterium*-mediated transformation has been enormously applied for gene functional studies, promoter characterization, production of pharmaceutically active compounds, elicitor identification, gene silencing, and CRISPR/Cas9-based genome editing, which facilitates the rapid improvement of crops. Although highly efficient somatic embryogenesis protocols are available, efficient genetic modification is lacking, which is a constraint in obtaining transgenic and genome-edited plants with desired agronomic traits. Of the four reports on the genetic modification of black pepper through *Agrobacterium*-mediated transformation, those by Sasikumar and Veluthambi [5] and Sim *et al*. [6] were unsuccessful in regenerating transformed plants. Varghese and Bhat [7] and Revathy *et al*. [8] emphasized the recalcitrance of black pepper, and their protocols enabled the establishment of only a few plants under greenhouse conditions. Agroinfiltration is a high-throughput fast-track strategy for evaluating disease resistance, pathogen responses, and crop disease defense mechanisms [9, 10]. Composite plants, that is, plantlets with transgenic roots (induced by *A. rhizogenes*) and wild-type shoots, are an elegant tool for developing plants expressing the gene of interest, especially those that enhance resistance to biotic stresses, and are also used in root biology studies. As black pepper is highly prone to root-devastating diseases, especially root-rot, the development of composite plants expressing the desired genes in the roots will reduce severe yield loss by root diseases and the uproar on transgenic food, as the peppercorns on the vines are nontransgenic. Bioreactors are useful for scaling up the production of plantlets, especially newly released black pepper cultivars through conventional breeding or genetic engineering, to meet the demand of farmers in a short period. Commercial-level production of plant saplings using temporary immersion bioreactors has been emphasized for many crops [11–13].

Our ultimate goal is to develop improved black pepper varieties tolerant to biotic (fungal and insect pests) and abiotic (drought, salinity, and heat) stresses through transformation and genome editing, which mandates a simple, fast, and high-throughput transformation protocol. Thus, in this study, we describe a sure-to-regenerate transgenic and genome-edited plant procedure through the *Agrobacterium*-mediated transformation system with an add-on of bioreactor scale-up production, agroinfiltration, and the development of composite plants of black pepper, which will serve as a lead for its improvement.

## Results

### Establishment of nucellar apomictic embryos

#### Primary somatic embryogenesis

**Figure 1:**
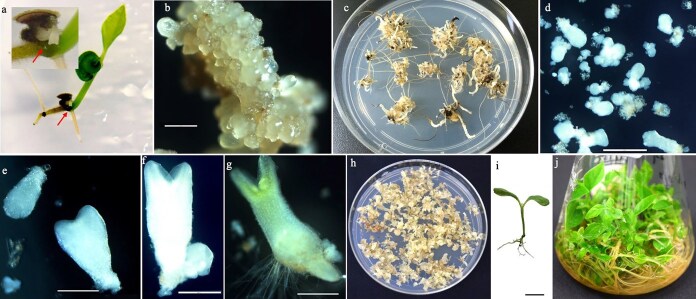
Nucellar apomictic somatic embryogenesis and plantlet regeneration. (a) formation of primary somatic embryo (arrows); (b–h) secondary embryogenesis; (b) globular embryos; (c) embryogenic cultures on SH solid medium with 1% sucrose in the dark; (d) embryos at early stages; (e) torpedo stage; (f, g) early cotyledonary embryo; (h) early cotyledonary embryos on SH solid medium with 3% sucrose in the dark; (i, j) Plantlets developed in SH suspension culture with 3% glucose. (Scale bars: b, d, e–g = 500 μm; i = 10 mm.)

Zygotic embryos from the cultured micropylar region of both varieties protruded within a week on SH basal medium with 3% sucrose (SHS3). Nucellus-derived embryos originated either attached at the root-shoot junction of the emerged zygotic embryos (Fig. 1a) or bulged out through the micropyle. Twenty and 22% of the explants of Karimunda and Sreekara produced somatic embryos, respectively, in 50 ± 10 days on SHS3 in the dark. The primary embryogenic potential of Karimunda and Sreekara was a mean of 5.7 and 4.8, respectively.

#### Secondary/cyclic somatic embryogenesis

Secondary embryos originated in the epicotyl region, that is, at the root-shoot junction or from the root pole of the primary somatic embryos (PSE) in 8–12 days of culture. Following the induction of a small embryogenic mass, numerous embryos emerged from these areas of the PSE, and the number of embryos increased further (Fig. 1b). The embryos progressed through different embryonal development stages, namely globular, heart, torpedo, and cotyledon, during the culture period (Fig. 1b–h).

The culture of the secondary embryos of both Karimunda and Sreekara and the culture of embryos in succession continued recurrently in a cyclic manner. Cyclic somatic embryos were observed at 65 ± 5 days in the dark. Media strength and sucrose concentration played critical roles in cyclic somatic embryogenesis of nucellar apomictic somatic embryos. Somatic embryos were white or pale yellow, appeared in clusters, and were friable (Fig. 1e and f). SH medium with 1% sucrose (SHS1) facilitated the proliferation of embryogenic calluses with a few late-stage embryos. The embryos were at different developmental stages, with the globular stage being predominant. Somatic embryos were developed from the globular stage onwards, in addition to the root pole of the torpedo stage. Occasionally, a few embryos progressed to the cotyledonary stage with distinct shoot and root poles in the same medium. Cotyledons remained white and exhibited the formation of new embryos on SHS1 in the dark (Fig. 1g). Suspension culture of the embryogenic calluses SHS1 displayed similar results, but with increased proliferation of embryogenic masses and embryos in the dark.

### Conversion of somatic embryos and carbon source

**Figure 2:**
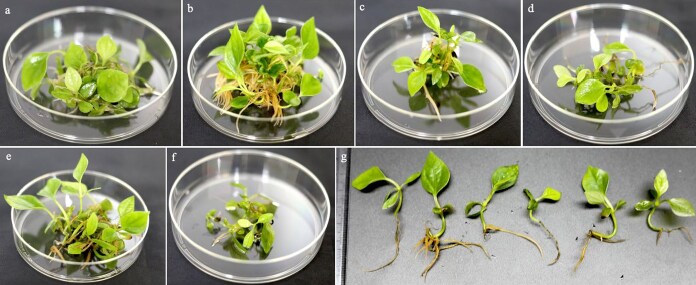
Conversion of somatic embryos and root growth in SH liquid media containing different sugars. (a) 3% sucrose, (b) 3% glucose, (c) 3% maltose, (d) 1.5% sucrose, (e) 1.5% glucose, and (f) 1.5% maltose. (g) Plantlets grown in SH liquid medium (left to right) 3% sucrose, 3% glucose, 3% maltose, 1.5% glucose, and 1.5% maltose.

**Figure 3:**
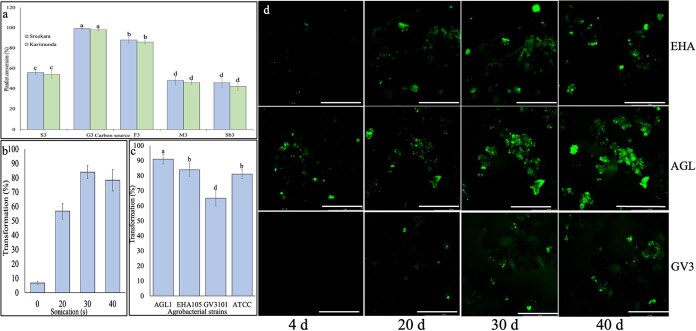
(a) Conversion percentage of somatic embryos in SH liquid media with different sugars (3%). (b) Sonication efficiency of *A. tumefaciens* -mediated transformation using EHA105. (c) Transformation efficacy of embryogenic calluses by *Agrobacterium* strains with 30 s sonication. (d) Expression of GFP on embryogenic calluses transformed with various *A. tumefaciens* strains at different days after infection. (a, c) Different letters represent the significant differences at 5% level (*P* ≤ 0.05). (b) All values are significantly different at 5% level (*P* ≤ 0.05; *t*-test). (Scale bars = 6 mm).

The sucrose concentration in the medium displayed embryogenic proliferation of both Karimunda and Sreekara. A sucrose concentration >1%, i.e. 1.5%, 2%, and 3% favored progression to the cotyledon stage, particularly at 3% (Fig. 1h), and they became green and converted to plantlets in the light (Fig. 1i and j). Regarding the increase in sucrose levels, the proliferation of embryogenic cells and early stage embryos, globular, and heart, in particular, was decreased. SHS3 promotes embryo maturation and conversion to plantlets. The use of glucose or maltose instead of sucrose resulted in significant differences in proliferation and embryo conversion. SHG1 facilitated embryo proliferation, and the SH medium with 3% glucose (SHG3) showed very little proliferation of the embryogenic mass compared to SHS3, which exhibited higher proliferation of embryos than glucose (Fig. 2a–g). The conversion of somatic embryos to plantlets in SHG3 was superior to that in sucrose or maltose. In the suspension culture of SHG3, the transfer of embryos in the early torpedo stages, which turned green, and the plantlet conversion was 100% over 83% in the SHS3 medium (Fig. 3a). Furthermore, in SHG3, the plants were greener and less hyperhydric, with a thick taproot (Fig. 2g), which later developed thick branches (Fig. 2g). Taproot development in the SHS3 medium was weak (Fig. 2g), necessitating subculturing on solid WPM (2% sucrose) medium containing activated charcoal (0.1%) to develop robust roots. Morphologically, plantlets developed in the SHG3 suspension medium under light were greener and healthier than those in SHS3 medium. The efficacy of plantlet conversion in fructose was similar to that in glucose, but the roots developed poorly and were very thin and slender. Plantlets formed in maltose- or sorbitol-containing medium (1.5% and 3%) were unhealthy, and root growth was inferior to that in fructose. In summary, root growth was superior in the glucose-containing medium, followed by that in the fructose-containing medium. The responses of both varieties, Karimunda and Sreekara, were similar with respect to the carbon sources.

### Osmotic potential of media with different carbon sources

SH media with various carbon sources at different concentrations, before and after the culture of 15 days, showed differences in osmotic potential (OP; Fig. S1). The OP of glucose at 1% after culture was higher than that of the media before culture (Fig. S1). At 3%, the OP was 2.3-fold less after embryo culture. In the case of 1% sucrose, the initial OP of the medium was higher, and after culture, it increased to 2.12-fold; at 3% sucrose, a 2.8-fold increase was noted. In the case of 3% fructose, the increase was 0.7-fold. Sorbitol and maltose at 3% concentration showed no fold change in OP before and after the culture.

### Sensitivity to antibiotics

Embryogenic masses cultured on SHS1 supplemented with lower concentrations of hygromycin (10 and 20 mg l^−1^) and kanamycin (25 and 50 mg l^−1^) survived (Fig. S1b and c). At 30 mg l^−1^ hygromycin, the embryogenic mass initially showed proliferation of embryos (10%), but to a lesser degree when compared to no or lower concentrations; later, brown and dead. Hygromycin at 100 mg l^−1^ displayed browning of the embryogenic mass in 7 days, later browned completely, and died. In the case of kanamycin, 25 mg l^−1^ exhibited proliferation of 40% of the embryogenic mass initially, but later browned, and at 100 mg l^-1,^ the initial proliferation was <10% with high browning and death in 25 days (Fig. S1b and c). The sensitivity of both Karimunda and Sreekara to the antibiotics was similar. Thus, the selection of the transformed tissues of both varieties was carried out by culturing initially on medium with 30 mg l^−1^ hygromycin/25 mg l^−1^ kanamycin for 15 days and transferring them onto medium with 30 mg l^−1^ for hygromycin and 50 mg l^−1^ for kanamycin, which facilitated a stringent selection of transgenic embryos.

### *Agrobacterium*-mediated transformation, selection, and transgenic plants regeneration

#### Effect of sonication

The transformation frequency of the embryogenic mass under different sonication treatments (20, 30, and 40 s) was significantly different from that of the control, which was not sonicated (Fig. 3b). Sonication resulted in a significant difference in the stable transformation frequency (Fig. 3b). The browning of untransformed embryos was observed after 14 days on the selection medium (SHS1 with 300 mg l^−1^ timentin and 25 mgl^−1^ kanamycin SHS1TK25)/hygromycin (SHS1TH30). The mean stable transformation frequencies for 0, 20, 30, and 40 s of sonication were 3%, 56%, 84%, and 78%, respectively (Fig. 3b). Sonication durations of 20 s and 40 s enabled GFP expression in individual embryos within the embryogenic mass. Although the transient expression at 40 s sonication was high, the emergence of embryogenic masses and globular embryos was low (Fig. 3b). Therefore, transformation experiments were carried out using 30 s sonication. The transformation frequencies of RFP and GUS at 30 s of sonication were 80% and 83%, respectively.

### Effect of infection and cocultivation media

Of the different infection and cocultivation media, the embryogenic calluses infected and cocultivated on full-strength SH and MS media as well as half-strength SH with 3% sucrose displayed very low expression of GFP after 4 days on cocultivation medium. Infection using half-strength MS medium with 30 s sonication and cocultured on half-strength MS with 3% sucrose exhibited high GFP expression (Fig. S1d), and was used in subsequent transformations.

### Efficacy of *Agrobacterium* strains

Among the different *Agrobacterium tumefaciens* strains, AGL1 displayed a higher stable infection efficiency than EHA105 and GV310. The transient expression of GFP was observed with AGL1, followed by EHA105, and GV3101 (Fig. 3c). Embryogenic calluses on the first selection medium (SHS1T300H30) were subcultured after 15 days onto SHS1T300H30. AGL1 showed the highest stable transformation efficiency (91%), followed by EHA105 (84%) and GV3101 (65%). Figure 3d shows the GFP expression of different agrobacterial strains from days 4 to 30 on the selection medium. The stable transformation efficacy of *A. rhizogenes* strains (81%) was not significantly different from that of EHA105 (Fig. 3c).

### Regeneration of transgenic plants

*Agrobacterium*-infected embryogenic calluses on the selection medium containing timentin (SHS1T300) with 25 mg l^−1^ kanamycin (SHS1T300K25)/hygromycin (SHS1T300H30) in the dark initiated growth as white spots. Globular-shaped embryos appeared after a week and progressed to the heart stage. The noninfected embryogenic mass turned brown (Fig. S2a–d). The transformed embryogenic masses were visibly distinguishable by their white/pale yellow color from the untransformed ones, which turned brown/black (Fig. S2a–d). The embryogenic masses individually subcultured after 15 days on fresh SHS1T300 with 50 mg l^−1^ kanamycin (SHS1T300K50)/hygromycin (SHS1T300H30) were proliferated well in the dark. A mean of 5.6 new embryos were observed on each mass within 10 days after transfer. The embryogenic mass and embryos proliferated well afterwards, and progressed through different stages to cotyledonary stages on this medium in 50–60 days. Cotyledonary embryos transferred into SHG3T100 (100 mg l^−1^ timentin) suspension cultures in the light were converted to plantlets with a mean of 5.3 roots per plant.

### Induction of hairy roots

**Figure 4:**
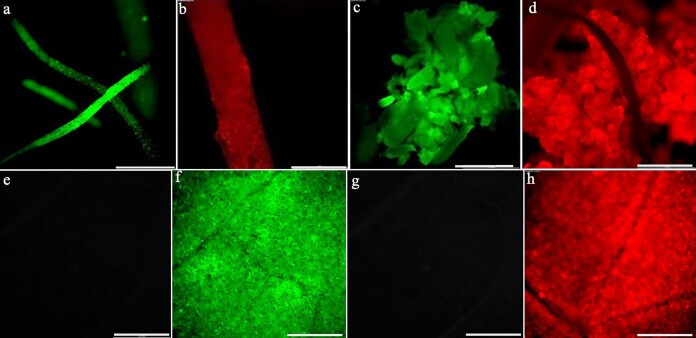
*Agrobacterium rhizogenes*-mediated transformation of black pepper embryogenic calluses. (a, b) Hairy roots expressing GFP and RFP, respectively. (c, d) Transformed embryos expressing GFP and RFP, respectively. (e–h) *Agrobacterium* infiltration of GFP and RFP into black pepper leaves, (e) WT under GFP filter, (f) GFP infiltrated leaf (g) WT under RFP filter, (h) RFP infiltrated leaf. (Scale bars: a = 2.1 mm; b = 775 μm; c = 3.3 mm; d = 1.8 mm; e, f, g, h = 1.2 mm.)

Infection of embryogenic calluses with *A. rhizogenes* induced hairy roots. The transformed roots were long and hairy (Fig. 4a–d). The hairy roots later induced an embryogenic mass with globular embryos on SHS1T300K25. Subculturing the embryogenic mass on fresh medium (SHS1T300K50) resulted in the proliferation of the embryogenic mass, progressing through different developmental stages to cotyledonary embryos. The transfer of late torpedoes to cotyledonary embryos in suspension cultures of SHG3T150 facilitated the conversion of 99% of embryos to plantlets.

### Agroinfiltration of black pepper leaves

Agroinfiltration of *A. tumefaciens* harboring binary plasmids containing *mgfp* or *rfp* into the leaves of black pepper showed the expression of GFP/RFP after 3 days (Fig. 4e–h).

### Generation of composite plants

**Figure 5:**
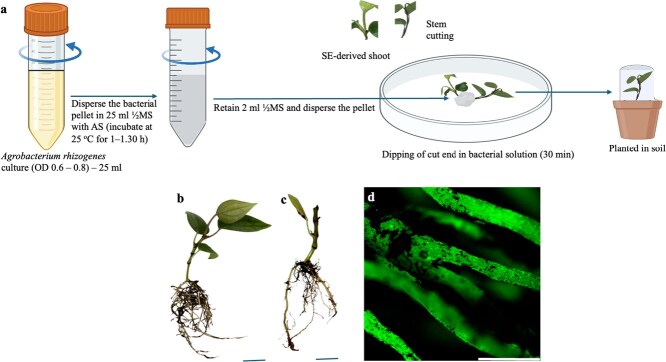
Composite plants of black pepper. (a) Schematic diagram of composite plants generation using *A. rhizogenes* (Biorender). (b, c) Composite plants of somatic embryo-derived established plants and stem cutting after 45 days, respectively. (d) Roots expressing GFP. (Scale bars: a, b = 5 mm, d = 2.3 mm.)

Figure 5a shows the schema of the composite plant generation. Root initiation from *the A. rhizogenes-*infected regions of the shoots was observed after 15 days. Root growth was initially slow and grew well 4 weeks after agroinfection (Fig. 5b and c). The transformation efficiencies of the hardened somatic embryo-derived plants and shoots of plants were 70% and 55%, respectively. The infected plants showed transformed (expressing GFP) and nontransformed roots (Fig. 5d). The hardened somatic embryo-derived plants and the shoots infected produced a mean of 8.3 ± 0.8 and 5.7 ± 0.4 roots, respectively. The hairy roots grew faster after 3 weeks than the nontransformed roots.

### Reporter genes expression

**Figure 6:**
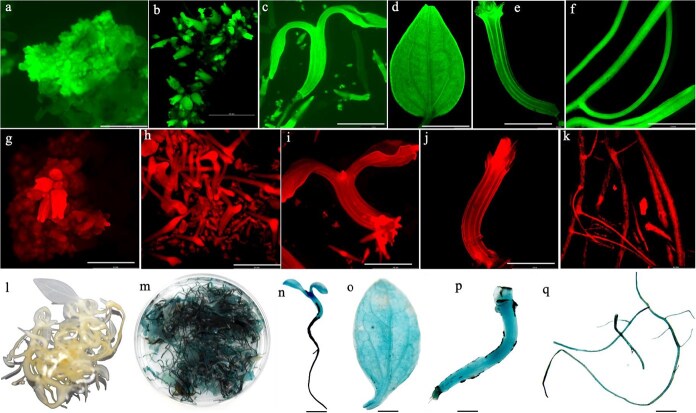
Expression of reporter genes in transformed tissues of black pepper. (a–f) expression of GFP: (a) embryogenic mass, (b) developed somatic embryos, (c) cotyledonary embryo, (d) leaf, (e) stem, (f) root; (a–f) expression of RFP: (g) embryogenic mass, (h) developed somatic embryos, (i) cotyledonary embryo, (j) stem, (k) root; (f) expression of GUS: (l) untransformed embryos, (m) somatic embryos, (n) cotyledonary embryo, (o) leaf, (p) stem, and (q) root. (Scale bars: a = 1.4 mm, b = 5.6 mm, c = 3.4 mm, d = 9 mm, e = 6 mm, f = 4.5 mm, g = 3.6 mm, h = 3.4 mm.)

Transformation was visually confirmed by the GUS assay and GFP/RFP expression as to the constructs used for transformation (Fig. 6a–q). Distinct GFP/RFP expression was observed from emerging embryogenic masses with somatic embryos. GUS histochemical staining and GFP/RFP expression were observed in different stages of somatic embryos (globular, heart, and torpedo-shaped), cotyledons, true leaves, petioles, roots, stems, and shoot tips (Fig. 6a–q). No GFP/RFP expression or GUS activity was detected in untransformed tissues (Fig. 6l). GFP and RFP were observed on hairy roots induced by *A. rhizogenes,* and the plants were regenerated through embryogenesis (Fig. 4a–d).

### Genome editing of *Pds*

**Figure 7:**
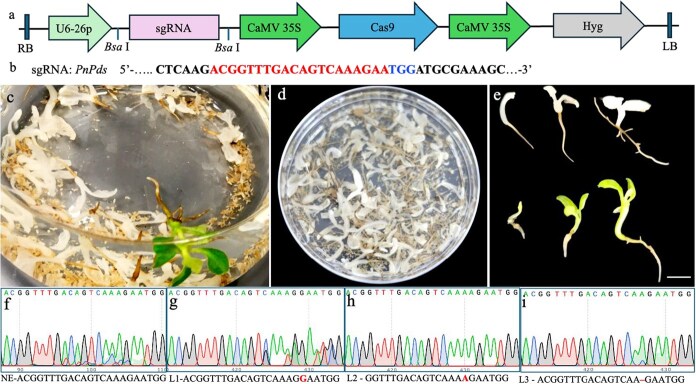
*PnPds* editing in black pepper. (a) Schematic representation of the pHSE401 vector. RB and LB, right border of T-DNA and left border of T-DNA, respectively; U6-26p, *Arabidopsis* U6 gene promoters; CaMV 35S, Cauliflower mosaic virus 35S promoter; Hyg, Hygromycin gene. (b) Bases in red and blue represents target sites and PAM sequences for the sgRNAs, respectively; vertical lines indicate *BSA I* restriction sites; (c-e) *Pds*-edited somatic embryo- derived plantlets exhibiting albino phenotype (SH medium with 3% glucose; Scale-1 cm); (f–i) chromatograms showing the editing (NE, non-edited; L1–L3 edited lines).

Figure 7a and b show schematic representations of the vector cassettes and sgRNA, respectively. Embryogenic calluses transformed with genome-edited constructs selected on medium (SHS1T300H30) appeared as white or cream spots on the embryogenic masses and showed proliferation. Subculturing of these proliferated tissues onto SHS1T300H30 progressed from globular to cotyledonary embryos. Cotyledonary embryos were converted to plantlets in SHG3T150. The genome-edited somatic embryos converted to plantlets appeared as albino. Non-edited plants were easily identified visually by their green phenotype, compared to the edited albino phenotype. The genome editing frequency was 89%, that is*,* 89% of the infected embryogenic mass-derived embryos showed an albino phenotype. The edited plants were short and had small leaves (Fig. 7c–e). The sequences of the amplified products of *Pds* using PnPDSFL-F and PnPDSFL-R (Table S1) from the individual edited lines displayed indels (Fig. 7f–i). The edited lines showed the insertion of either base G or A and deletion of base A within the target (Fig. 7f–i). All edited lines exhibited albino phenotypes. Analysis of the ORFs of the insertion mutant lines showed an in-frame premature stop codon protein at 51 b of the coding region, whereas the deletion mutant resulted in an alteration of the amino acid sequence.

### Plantlet establishment

**Figure 8:**
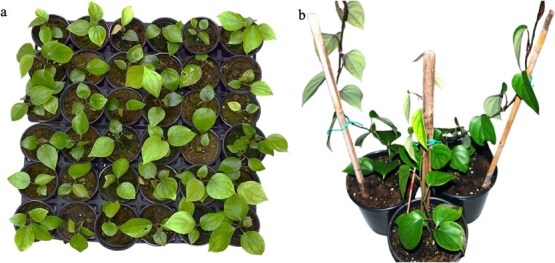
(a, b) Acclimatized transgenic plants, 30 and 90 days after hardening, respectively.

Plantlets transplanted directly from the SHG3 liquid medium under light conditions exhibited 99% survival after hardening, and the plants grew well (Fig. 8a and b). The plantlet survival rate from SHS3 after subculturing on half-strength MS solid medium with activated charcoal was 53%. Both varieties displayed no significant differences in terms of plantlet survival. Transgenic plants produced in SHG3 medium showed 99% survival rate in soil establishment.

### Genotyping of transgenic plants and Western blot

PCR analysis confirmed the presence of transgenes in the 10 independent transformed plants of each construct by the detection of 353, 753, 681, and 753 bp amplified bands corresponding to *gusA*, *mgfp, dsRed,* and pREDROOT genes, respectively (Fig. S3a–e). Transgenic roots of composite plants were confirmed by PCR using GFP-F and GFP-R primers (Table S1; Fig. S3b). Western blotting of proteins from the six GFP-positive lines exhibited a single band of ~28 kDa (Fig. S3f).

### Relative expression studies

**Figure 9:**
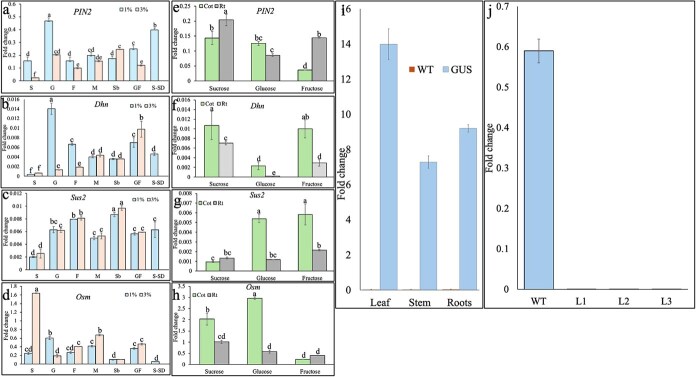
Expression of different genes by qPCR. (a–d) expression of genes at 1% and 3% carbon sources in somatic embryos; (e–h) expression in cotyledons and root at 3% carbon sources; (i) expression of GUS in stem, leaf, and root of plants transformed with gusA; (j) Expression of *Pds* edited lines. ( All values in (i) and (j) are significantly different at 5% level (*P* ≤ 0.05; *t*-test). Different letters in (a)–(h) shows significant difference at 5% level (*P* ≤ 0.05; DMRT).

Gene(s) expression analyses during embryo maturation/conversion showed that glucose is the superior carbon source. *PnPIN2* showed the highest expression in liquid and on solid media containing 1% glucose and was lower in 3% liquid media. Fructose at 1% followed 1% glucose (Fig. 9a). The expression profile of *PnDhn1* (LEA1) exhibited a trend similar to that of *PIN2*, with the highest expression at 1% glucose (Fig. 9b). The expression of *PnSus2* was highest at 3% sorbitol, followed by 3% fructose (Fig. 9c). In the case of *PnOsm*, the highest expression was observed at 3% sucrose, followed by 1% glucose, and the lowest was on solid media containing 1% glucose (Fig. 9d). There was no significant difference in expression between 1% (solid and liquid) and 3% glucose media. Media with 1% and 3% sucrose displayed lower expression (Fig. 9a–d). Analysis of these genes at 3% carbon sources (glucose, sucrose, and fructose) in cotyledons and roots separately displayed differential expression (Fig. 9e–h). *PIN2* expression was the lowest in roots at 3% glucose and was highest at sucrose, followed by that at fructose (Fig. 9e). Of the cotyledons, the lowest expression was noted at 3% fructose, followed by glucose, and the highest was sucrose. The expression of *Dhn1* was higher in sucrose and fructose, but not significantly different; it was the lowest in glucose and significantly different from that in sucrose and fructose (Fig. 9f). The roots displayed the same trend, but all were significantly different from each other. The lowest value was observed for sucrose, which was significantly different from those of glucose and fructose. The expression of *Sus2* in fructose was highest in the roots, and the sucrose and glucose levels were lower, with no significant differences (Fig. 9g). In cotyledons, glucose and fructose exhibited the highest expression, with no significant difference between them, while sucrose exhibited the lowest expression, which was significantly different from that of glucose and fructose (Fig. 9g). OSM expression was highest in cotyledons at 3% glucose, followed by sucrose (Fig. 9h). The highest expression was observed in the roots treated with sucrose, followed by glucose. In the case of fructose, roots showed higher expression than cotyledons (Fig. 9h). The cotyledons of the embryos grown in 3% sucrose (not significantly different) showed the highest expression, followed by fructose (insignificant), and the lowest expression was observed in glucose (significantly different) (Fig. 9e–h). The expression levels *of PIN2, Osm, Sus2,* and *Dhn* in roots at 3% glucose were lower (also their difference and ratio in fold change between root and cotyledon) than those in sucrose and fructose (Fig. 9e–h). In addition, except in the case of *Osm,* the total fold change of cotyledons and roots in glucose was not significantly different from that observed in the whole embryos in 3% glucose. The differences in the fold change of the above genes in cotyledons and roots and their respective root: cotyledon fold-change ratios are provided in Table S2.

Relative expression of *gusA* in plants transformed with the *gusA* construct showed 13.9-fold, 7.2-fold, and 9.1-fold expression in leaf, stem, and root tissues of *gusA-positive* plantlets compared to that in wild-type plants (Fig. 9i). The expression of *Pds* in the edited lines was relatively low in all the lines tested compared to the high expression in non-edited plants (Fig. 9j).

### Bioreactor scale-up

**Figure 10:**
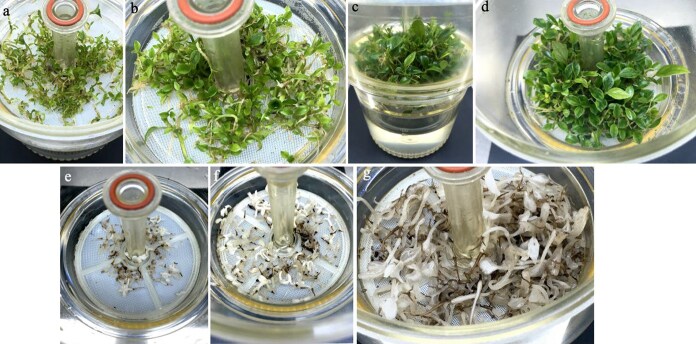
Different growth stages of transgenic plants (a–d) and *Pds* edited plants (e–g) in Temporary Immersion Bioreactor.

The rate of proliferation of embryogenic masses with the development of embryos to early or cotyledonary stages in SHS1 was twice that in TIB compared to that in suspension cultures. Replacement of the same medium after 21 days continued the proliferation of embryogenic masses and embryos until the cotyledonary stage. Changing the medium from SHS1 to SHG3 facilitated germination and plantlet growth. Germination of the untransformed and transformed embryos began on the fifth day after the change to SHG3 (Fig. 10a–d). Healthy plantlets were first harvested in the second week onwards to facilitate the growth of the remaining plantlets. The survival rate of the TIB system-derived plantlets was 99%. Compared to the suspension cultures in 250 ml conical flasks, the plants were less hyperhydric, healthy, and green. The growth of the plantlets was controlled by the duration of immersion in the TIB system, and the optimal time was 5–10 min per day. The TIB system was superior to the cultures in conical flasks, particularly in terms of plant proliferation and growth. In the TIB system, the plantlets were easily separable, unlike in the conical flask, where the plants were clumped with entwined roots. The untransformed, GFP, and *Pds-*edited lines (Fig. 10e–g) in the TIB system were similar, except for the plantlet growth of the edited lines, which was slow. The TIB system enabled the periodic harvest of plantlets from 2 weeks for hardening. A mean of 300 plantlets were produced in a three-month period from a single TIB system.

**Figure 11:**
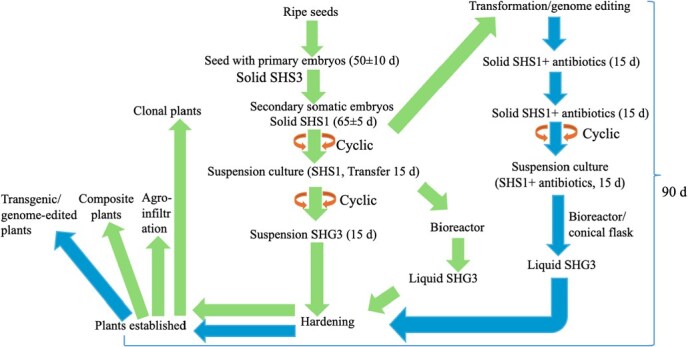
Schematic nutshell of the present (green and happy blue represents untransformed and transformed, respectively).

Figure 11 shows a schematic overview of the present study emphasizing transgenesis/genome editing, which requires 90 days to establish genetically modified plantlets in soil from transformation.

## Discussion

Nucellar apomixis-derived progenies arise without fertilization and meiosis, inherit the mother plant genotype, and preserve hybrid vigor for multiple generations in economically important plant genotypes [14–16]. *In vitro* nucellar apomixis is a high-throughput strategy for producing plants to meet the demand for genetically uniform disease-free plants in a short time and has been well reported, including different varieties of black pepper [3, 4, 17, 18]. The clonal fidelity of nucellar somatic embryo-derived plants using RAPD and SSR markers, a characteristic mainly due to maternal origin, in a PGR-free medium has been emphasized previously [4, 7]. Clonal propagation of black pepper through vegetative cuttings from elite clones is time-consuming. *In vitro* clonal propagation through shoot tips and node segments from different black pepper varieties has been reported [19, 20], but the rate of multiplication is a constraint to meet farmers’ demands in time. Clonal cyclic or recurrent somatic embryogenesis potential from primary embryos through secondary and tertiary embryogenesis is an elegant tool for improving crops, including black pepper, through genetic modification.

The survival percentage of plantlets determines the success of micropropagation and the price of saplings, especially for crops with high demands. In this study, the carbon source was key in determining the survival percentage of the nucellar apomixis-derived plantlets, which in turn relied on the conversion of embryos to healthy plants with well-developed taproot/root systems. The influence of carbon sources on cell division and differentiation is crucial for the progression of different phases of the plant cell cycle [21, 22]. The efficacy of carbon sources in somatic embryo induction, proliferation, maturation, and germination has been documented in blue spruce [23], cactus [24], walnut [25], potato [26], and alfalfa [27]. The effectiveness of different carbohydrate sources and their osmotic effects can vary depending on the plant species, genotype, and specific stages of somatic embryogenesis [28]. The water potential of normal plant cells in tissue culture media is typically more negative. Sucrose and maltose (disaccharides) generally have a higher OP than glucose, which lowers the water potential more than a similar solution of glucose or maltose [29]. PGR-free SH media containing different sugars were used in this study, which is advantageous considering the lack of PGR modulation. The OP of glucose at 1% after culture was higher than that of the media before culture. At 3% glucose, OP was 2.3-fold lower after embryo culture. In the case of 1% sucrose, the OP of the medium was higher, and after culture, it increased by 2.12-fold. At 3% sucrose, there was a 2.8-fold increase. At 3% fructose, the increase was 0.7-fold. Sorbitol and maltose at 3% concentration showed no fold change in OP before and after the culture. SHS1 (1% sucrose) solid media facilitated cyclic embryogenesis and the progression of somatic embryos to the cotyledonary stage. SHS3 (3% sucrose) disrupted the cyclic nature of somatic embryogenesis, favoring conversion to plantlets, but at a lower frequency. SHG3 (3% glucose) converted the embryos into healthy plants with well-developed taproot/root systems by limiting embryogenic proliferation. The conversion efficacy followed the order glucose > fructose > sucrose. SHM3 (3% maltose) and SHSb3 (3% sorbitol) were inferior to glucose, fructose, and sucrose. In the present study, healthy root (tap and adventitious) development differentiated the efficacy of the carbon sources. Glucose modulated the growth of shoots and roots, followed by fructose. All other carbon sources in our study promoted shoot growth over root growth. Sucrose was inferior to glucose and fructose, both individually and in combination, in terms of root growth. In *Asparagus officinalis,* glucose promoted root growth, fructose promoted shoots, and sucrose promoted both shoot and root growth [30]. Glucose significantly influences plant root growth and development by acting as both an energy source and signaling molecule, affecting root length and lateral root formation [31]. The efficacy of glucose as a carbon source and osmotic regulator for somatic embryogenesis over other sugars has been reported [32]. According to Stitz *et al*. [33], the breakdown of glucose in the pericycle, the outermost cell layer of the root cylinder, is critical for lateral root formation.

As a primary energy source, glucose is absorbed faster by plant cells than sucrose, maltose, fructose, and sorbitol because of its simple structure and direct availability as an energy source that contributes to osmotic regulation in the culture medium [34]. Glucose, a monosaccharide, can be directly absorbed by the cell membrane and metabolized, whereas sucrose, a disaccharide, must be hydrolyzed into glucose and fructose (structural isomer of glucose) before absorption [35]. Sucrose contains more energy per unit than glucose, and its nonreducing nature makes it more stable; it can be transported and stored for longer periods than glucose [35, 36]. Unlike sucrose and maltose (which require hydrolysis by maltase), glucose and fructose absorption by plant cells generally does not require direct energy and relies mainly on facilitated diffusion mechanisms. However, glucose absorption is faster than fructose absorption, primarily because of the differences in the transport mechanisms and metabolic pathways of these two sugars [37]. Glucose is a key substrate for glycolysis (a metabolic pathway that breaks down glucose into pyruvate) and other pathways, whereas fructose is converted to glucose or glucose-6-phosphate, which then enters the glycolytic pathway [38]. In agreement with Kyriazis *et al*. [39], the use of equimolar concentrations of glucose and fructose did not lead to the same signaling effects as sucrose, highlighting the glucose-specific signaling roles. It ensures intact delivery for signaling and energy transfer compared to glucose and can be rapidly taken up and utilized by cells or undergo various metabolic conversions [35, 36]. According to do Nascimento *et al*. [40], increasing sucrose concentrations to a certain point (e.g. 175 mM) can increase the number of mature somatic embryos. Inhibition of carrot somatic embryo radicle elongation by sucrose at or above 5% (145 mM) and its reversal by lowering the amount of sucrose have been reported [41]. They observed no inhibitory role of monosaccharides and inferred that the sucrose signaling pathway may be independent of the hexokinase pathway. In line with their observations in carrot somatic embryos, higher levels of glucose/fructose/maltose (3%) in our study were unfavorable for maintaining the embryonic status, especially cyclic somatic embryogenesis. As in their studies on carrot, black pepper embryos in glucose media developed more quickly, showing earlier initiation and faster radicle growth than fructose. Krook *et al*. [37] reported that fructose absorption and metabolism differ from those of glucose in carrot tissues. In contrast to their results, radicle growth in a medium with a combination of glucose and fructose was not favored as glucose alone. Sucrose also influences the timing and quality of somatic embryo maturation. Cao *et al*. [42] reported that sucrose is slightly superior to maltose in promoting the yield of stage 3 (torpedo stage) embryos. The superiority of maltose over sucrose in promoting somatic embryo development and maturation has been previously demonstrated [27]. Sorbitol, a sugar alcohol, is not easily utilized and requires more processing, making its absorption slower; however, its efficacy in the germination of somatic embryos has been reported [43].

Both sucrose and glucose play important roles as signaling molecules; however, sucrose is generally considered a more efficient and stable signaling molecule for long-distance transport and various developmental processes, such as phloem development and embryonic cell division [35, 44]. Glucose is generally considered a stronger and more versatile signaling molecule in plants than fructose, particularly in its direct role as a major carbon and energy source, with a well-established role in regulating gene expression, protein expression, and developmental programs from embryogenesis to senescence [45]. Glucose is involved in hexose-dependent signaling pathways that are sensed by external and intracellular receptors [46]. It has been reported that miR156 is a central factor in the regulation of the juvenile-to-adult transition, and an increase in sugar levels promotes miR156 downregulation, triggering the juvenile-to-adult transition [47, 48]. This juvenile-to-adult phase transition is thought to occur following an increase in glucose or a related metabolite (such as trehalose) beyond a threshold level [49].

PIN-FORMED (PIN) proteins, which are primarily involved in auxin transport, are modulated by various internal and external signals that influence plant development [50]. In the present study, somatic embryos in SH medium with 1% glucose showed the highest expression of *PIN2*. *PIN* genes, particularly *PIN2*, are responsive to glucose levels, with glucose signaling through TOR playing a role in regulating *PIN2* stability and function, thereby influencing auxin transport and root development [51, 52]. In the presence of glucose, TOR is activated, which phosphorylates and stabilizes PIN2, promoting auxin transport away from the elongation zone and allowing proper cell growth [51]. According to Leitner *et al*. [53], gravity signals trigger the differential ubiquitylation of *PIN2*, which may feedback on the coordination of auxin distribution in the root meristems. Although equimolar osmotica treatment increased the osmotic pressure in the medium, it did not facilitate radicle growth and indicated the activation of *PIN* by glucose rather than osmotic stress.

*Dhn* (dehydrin) is a specific class of LEA gene that encodes proteins that protect plants from environmental stresses. Glucose signaling has been reported to be involved in plant responses to various stresses at the molecular level by regulating the expression of LEA and ABA genes [54]. Glucose-mediated LEA gene activation is crucial for the maturation and acquisition of desiccation tolerance in somatic embryos [55]. The expression of *Dhn1* in the present study was highest in somatic embryos grown in 1% glucose, that is, at maturation. The stimulation of high expression of LEA genes with respect to increased glucose levels during the later stages of embryogenesis, potentially through signaling pathways involving ABA and other transcription factors, has been emphasized [56]. Once desiccation tolerance is acquired, as LEA gene expression is a part of a larger regulatory network that orchestrates the maturation and germination of somatic embryos, which ultimately leads to the conversion of plantlets [57]. LEA gene expression indicates the superior effect of glucose over other carbon sources in the germination of nucellar apomictic embryos of black pepper. The acquisition of optimal desiccation tolerance due to the faster uptake of glucose and OP of the medium may be the reason for the high conversion rate of the embryos to plantlets in our study.

In this study, the expression of *Sus2* was higher in glucose media than in sucrose and was highest in sorbitol media. This indicates that the expression in glucose media is optimal compared to that in sucrose (lower) and sorbitol (higher), which is inferior in conversion to plantlets. Studies have shown that increased glucose levels can lead to increased SuSy gene expression. The upregulation of *Sus1* expression is regulated by an HXK-dependent pathway [58]. Glucose produced during photosynthesis can be converted to sucrose, and SuSy can also play a role in this process by utilizing UDP-glucose formed from sucrose cleavage to synthesize sucrose [36]. In our study, the sucrose-supplemented medium showed the lowest expression of *Sus2.* In addition, the expression of *Osm* was higher in sucrose-supplemented medium than in glucose, maltose, glucose, sorbitol, or a combination of glucose and fructose. Our results are consistent with those of Yang *et al*. [41], who observed that osmotic pressure is not the only key factor regulating the elongation of somatic embryo radicles in carrot somatic embryos. The sucrose transporter *Suc2* is reportedly active in the presence of sugars, particularly in the context of phloem loading, and is crucial for transporting sucrose from source (such as leaves) to sink (such as developing fruits or roots) tissues [59].

The comparison of the differential expression of *PIN2, Osm, Sus2,* and *Dhn1* in roots and cotyledons at 3% glucose and their ratio in fold change between roots and cotyledons supports the points discussed above. The expression of *PIN2* was higher in the roots of embryos in sucrose and fructose and was highest in the cotyledons in the presence of glucose. Higher expression of *PIN2* in roots in sucrose reduces root growth, possibly by the inhibition of either higher levels or reduced catabolism. *Osm* and *Sus2* were expressed at higher levels in cotyledons in glucose than in sucrose and fructose, and in the case of root growth in glucose medium, this denotes the significance of the ratio of their expression in the cotyledons and roots. Although osmotic pressure and the expression of *Osm, Suc2,* and *Dhn* are involved in radicle elongation with respect to glucose, *PIN2* seems to be the leader in radicle elongation of black pepper, which facilitates higher plantlet establishment. This opens the window to explore glucose signaling in comparison to sucrose during the maturation and germination of somatic embryos. Transcriptome analysis will reveal the gene networks involved in somatic embryo conversion and interpret biological processes at the biochemical and molecular levels.

A roadmap of high-efficacy stable transformation is significant in recalcitrant crops to improve them by the integration of trait-regulating genes and to study their functional attributes. The single report of black pepper transformation efficiency is very low, with only eight plants expressing *gusA*. Various factors determine the efficiency of *Agrobacterium*-mediated plant transformation. In the present study, the infection medium, sonication, vacuum infiltration, and cocultivation duration were assessed, and the highest transformation was observed with half-strength MS medium (3% sucrose) for infection, 30 s sonication, 10 min vacuum infiltration, and a cocultivation period of 4 days on half-strength MS medium (3% sucrose) with AS. Sonication was the major factor affecting transformation efficacy. SAAT has been proven to be an ideal T-DNA delivery method for a number of plant crops, especially those that are relatively recalcitrant to transformation [60–63]. Although 40 s of sonication produced higher transient expression, the stable transformation efficacy decreased compared to 30 s, and this is assumed to be due to damage to the tissue and set back to revive. The efficiency of different agrobacterial strains depends on the plant genotype/tissue [64]. In this study, we used different strains and plant transformation vectors to assess agrobacterial infectivity. Our results displayed remarkable consistency and reproducibility of transformation efficiencies over six vectors (including genome editing, pHSE401) and various agrobacterial strains. Among the different strains, AGL1 exhibited the highest stable transformation efficiency, followed by EHA105, whereas GV3101 exhibited the lowest. The ability of agrobacterial strains to successfully transform plant cells is governed by their chromosomal genomes and plasmids, which collectively encode the requisite components for adhesion to plant cells and subsequent transfer of T-DNA [65]. It is pertinent to note that all three strains are derivatives of the C58 strain, which originates from A281. Both AGL1 and EHA105 harbor the hypervirulent pTiB0542 Ti plasmid, which carries additional virulence genes, whereas GV3101 harbors the Ti plasmid pmp90 (pTiC58DT-DNA). This genetic distinction likely accounts for the observed variations in transformation efficiency. Previous reports on black pepper by Varghese and Bhat [7] and Revathy *et al*. [8] were conducted using the EHA105. The efficacy of different agrobacterial strains has been demonstrated in several plant species, including AGL1 in cannabis [66], EHA105 in *Jatropha curcas* [67], soybean [68], and GV3101 in *Bacopa monnieri* [69]. PCR of different transgenic events expressing different reporter genes and western blotting of GFP confirmed the efficacy of the transformation. Relative *gusA* expression using qRT-PCR indicates the expression of genes of interest and functional studies. Transformation using *A. rhizogenes* is worthwhile, and regeneration facilitates the development of plants with genes specifically expressed in the roots under root-specific promoters. This allows the development of transgenic plants to compete against root diseases in black pepper, especially root-rot caused by *P. capsici*.

Antibiotic dosage is critical for the selection and recovery of transformed tissues in black pepper. Initial high doses were efficient for selection but damaged transformed tissues with GFP expression. The initial low dose for 15 days and subsequent transfer to a high dose mitigated the initial detrimental effects of the high dose after cocultivation and enabled stringent selection.

Agroinfiltration of *mgfp* into the leaves of somatic embryo-derived plants in this study resulted in GFP expression. Agroinfiltration is a widely exploited technique used in plant biology to achieve a quick expression profile of genes infiltrated into leaf cells transiently. Agroinfiltration in black pepper is highly relevant for quickly assessing the interactions of pathogen-related genes and their resistance, as the crop is highly sensitive to several pathogens, particularly, fungi. In addition, it has potential in virus-induced gene silencing studies of black pepper, especially that of *Piper yellow mottle virus* and *Cucumber mosaic virus* (CMV), the most damaging viruses of this crop.

Plants with transformed (hairy) roots and a nontransgenic shoot, well known as composite plants, curtail many of the biosafety concerns related to transgenic plants. Hairy roots of composite plants harboring genes for biotic tolerance can efficiently compete against root-specific pathogens, which severely affect crop yields. Black pepper is highly prone to root-devastating diseases, and crop loss, especially due to root rot, is high. Root-rot destroys the root system, impacting the plant’s ability to absorb water and nutrients, thereby affecting crop yield. The loss is estimated to be 10%-15% in some regions and up to 95% in severe cases. Alegbejo *et al*. [70] reported a revenue loss of USD 1700-USD 3200 per hectare. Attempts to reduce root diseases through biological control have been reported to be only partially successful. The development of composite plants expressing the desired genes in the roots to compete against diseases will reduce the severe yield losses of black pepper. This method not only allows for stable gene expression but also provides a transient system for studying root biology, plant-microbe interactions, gene function, metabolic pathways, and root development dynamics. Composite plants, mostly with marker genes, have been reported in different crops with varied transformation efficacy, including cucumber [71], pea [72], chickpea [73], Chinese cabbage [74], and soybean [75]. Ma *et al*. [76] accomplished genome editing through hairy roots from stem cuttings of citrus genotypes. We obtained composite black pepper plants with >60% transformation efficiency using somatic embryo-derived plantlets and stem cuttings in a single step. The accomplishment of nucellar apomixis-derived clonal plants at the bioreactor level with 99% survival enables the production of large-scale composite (clonal) plants with the desired trait gene in a short time. As black pepper is propagated mainly through stem cuttings, this method is a practical way to introduce new traits, such as disease resistance, and improve the productivity of black pepper. Hairy roots are highly branched, and it facilitate efficient nutrient uptake, which reflect in the yield.

Disruption of phytoene desaturase (*Pds*), which plays a vital role in chlorophyll biosynthesis, is usually explored as a visual marker for genome editing in many plant species. Knockout of *Pds* causes the impairment of chlorophyll biosynthesis, resulting in the development of plants lacking chlorophyll, appearing mosaic or albino, a phenotype that visually confirms the efficiency and specificity of CRISPR/*Cas9* editing. CRISPR/Cas9 is considered the most effective genome-editing tool and has been efficiently applied for trait improvement in several crops, such as tomato and rice. We successfully edited *Pds* with 89% efficiency, and this is the first report of genome editing in black pepper. All edited lines displayed an albino phenotype due to an in-frame premature stop codon in the ORF of *Pds.* The slow growth of edited lines is due to the disruption of *Pds*, impairing the chlorophyll, carotenoid, and gibberellic acid pathways, which has also been reported in *Arabidopsis* [77]. The high-efficiency editing described in the present study paves the way for editing black pepper to improve the desired traits.

The transgenesis/genome editing procedure in our study required 3 months from transformation to establish genetically modified plants in soil. The maintenance of the nucellar embryogenic masses for longer periods (3+ years) after establishment using SH basal medium serves as a time saver for the improvement of black pepper using genetic modification, especially genome editing. Cyclic embryogenic potential enables the production of transformed/edited plants on a large scale on demand, even by scale-up through bioreactor systems, as demonstrated in this study. The stringent roadmap of highly efficient and reproducible transformation with respect to different strategies, such as transgenesis, hairy root induction, and composite plants of black pepper in this study, further proved by high-frequency genome editing through CRISPR/Cas9, is a breakthrough to accelerate black pepper improvement programs.

Bioreactors enable the production of high-volume plantlets with minimal human interference. The scale-up of plantlet production through *in vitro* culture using different types of bioreactors has been accomplished in several commercially important species [11–13]. Bioreactors are useful for scaling up plantlet production using high-proliferating somatic embryos, especially through nucellar apomixis, which assures quality clonal plant saplings to farmers. Pilot-scale production of nucellar apomixis-derived plants and genetically modified (transgenic and genome-edited) plants in our study is advantageous for providing plantlets at low prices in a short timeframe.

## Conclusions

The improvement of black pepper fundamentally requires the use of various methods. The present study offers multifaceted applications for the improvement of the highly valuable spice crop, black pepper. The highly efficient transformation strategy, hairy root induction, agroinfiltration, development of composite plants, and genome editing serve as tools to complement the improvement of black pepper. The development of transgenic somatic embryos with GFP/RFP-tagged genes of interest is promising for studying the significance of genes in the conversion of somatic embryos concerning carbon sources. The bioreactor scale-up of nucellar apomixis-derived plants, transgenic, and edited plants, with the adoption of automated large bioreactors, enables the delivery of plants at a low cost to farmers, which boosts crop yield to meet the escalated demand for peppercorns. In addition, transcriptome studies of carbon source-driven induction and germination of nucellar somatic embryos in a medium without hormonal stimuli provide new insights into germination and orchestration of genes at the molecular level. In summary, the present study promises to improve this valuable crop.

## Materials and methods

### Seeds surface sterilization

Fresh ripe seeds of black pepper (*P. nigrum* L.) varieties Karimunda (the ancestor pepper) and Sreekara (collected in January 2022), after removing the skin, were surface disinfected in a 250 ml Erlenmeyer flask by immersing in 50 ml of 20% (v/v) commercial bleach solution with 0.1% (v/v) Tween-20 for 20 min. Subsequently, the seeds were washed five times (5 min each) with sterile water and dried on sterile filter paper. The micropylar domes with the nucellar tissue (explant) scooped out from the disinfected seeds were cultured on Schenk and Hildebrandt [78] (SH) basal solid medium (Phytotech Labs, USA) containing 3% (w/v) sucrose and incubated in the dark at 25 ± 1°C.

### Initiation and maintenance of callus

Nucellus-derived embryogenic callus was initiated and subcultured according to the protocol of Nair and Gupta [3] and Sasi and Bhat [4]. The primary somatic embryos formed on the scooped micropylar region were subcultured on basal SH with 1% (w/v) sucrose and incubated in the dark for secondary and cyclic somatic embryos.

### Plantlet regeneration from somatic embryos and acclimatization

Conversion of somatic embryos to plantlets was attempted using SH medium with different levels (1%-3%, w/v) of carbon sources: sucrose, glucose, fructose, maltose, sorbitol individually, and a combination of glucose and fructose (0.5 and 1.5% each). Somatic embryogenic masses (~50 mg) were transferred into 50 ml SH liquid media in 250 ml Erlenmeyer flasks containing different carbon sources and incubated on a rotary shaker (Eppendorf, Hamburg, Germany) under a 16 h light/8 h dark photoperiod. Each treatment was performed in triplicate and repeated thrice. The liquid medium was replaced with fresh medium after 15 days. Plants with poorly developed roots were transferred to Woody Plant Medium [79] (WPM; Phytotech Labs, USA) with 0.1% (w/v) activated charcoal for better root growth. After 30 days of culture, the conversion of embryos into plantlets was examined and transferred to a soil mix of sand and potting soil (1:1) in small pots (4 cm × 4 cm). The pots were covered with a moistened transparent plastic polyethylene bag and acclimatized in the plant room (25 ± 2°C, 70% humidity, 16 h light: 8 h dark photoperiod, 400 μmol m^−2^ s^−1^ - Heliospectra LED lights). The cover was removed after 15 days, and the plants were subsequently transplanted into 5 l pots (21 cm w × 19 cm ht) and grown in the plant room.

### Determination of OP

The OP of SH media containing 1% and 3% of sucrose, glucose, maltose, fructose, sorbitol, and combinations of glucose and fructose (0.5% and 1.5% each) at 25°C was determined before (after autoclaving) and 15 days of culture (equal amounts of somatic embryos) as described by Liu and Chi [80] using the formula OP (kPa) ≈ −50 × EC (dS m^−1^) after measuring with a conductivity meter (TDS Conductivity Meter, USA).

### Determination of hygromycin and kanamycin sensitivity

Antibiotic sensitivity for selecting the transformed embryogenic calluses was determined by culturing the untransformed embryogenic calluses on basal SHS1 solid medium containing various levels of hygromycin (10, 20, 30, 50, 75, and 100 mg l^−1^) and kanamycin (25, 50, 75, and 100 mg l^−1^) along with 300 mg l^−1^ timentin in the dark. Two plates of calluses were used for each dose of hygromycin and kanamycin, and the experiment was repeated thrice.

### *Agrobacterium* strains and plasmid vectors

Different strains of *A. tumefaciens* (EHA 105, AGL1, and GV3101) and *A. rhizogenes* (ATCC15834) were used in this study. Binary vectors pH7m24-35S:*mGFP* and pCAMBIA1201 [60] (Prof. Eduardo Blumwlad, University of California, Davis, USA), pREDROOT (with RFP), p35S:*mgfp* (*gusA* of pCAMBIA2301 replaced with *mgfp*), and p35S:*dsRed* (*gus^+^* of pCAMBIA1305.2 replaced with *dsRed)* were used for the present study. The features of transformation vectors and culture of different agrobacterial strains on media plates with the respective antibiotics have been described in our previous studies [61, 81].

### *Agrobacterium* culture

Cultures of *A. tumefaciens* strains and *A. rhizogenes* for transformation were grown and prepared as detailed in previous studies [61, 81] with specific antibiotics until incubation of the bacterial cultures at 50 rpm for 1–2 h at room temperature (RT; 23°C-025°C) in the dark.

### Sonication, infection, and cocultivation medium

The transformation parameters, such as the infection medium, sonication, and cocultivation medium, were optimized using ~400–500 mg embryogenic calluses with the EHA 105 strain harboring a binary vector with *mgfp*. Four infection (liquid) and cocultivation media were used: half- and full-strength SH and half- and full-strength Murashige and Skoog (MS) media [82]. All media were supplemented with 200 μM acetosyringone (AS). The optimum sonication time was determined by sonicating the embryogenic calluses at 60 kHz for 0, 20, 30, and 40 s in 25 ml of bacterial suspension in a 50 ml Falcon tube using a bath-type sonicator (Branson 5800, USA). Sonicated embryogenic calluses were incubated at 50 rpm for 20 min at RT (23°C-25°C) in the dark, followed by vacuum infiltration (10 kPa for 10 min). The infection medium was decanted by pouring or using a pipette. The infected calluses were spread on double-layered sterile filter paper in a Petri dish to blot excess bacteria. The dried infected calluses were spread on filter paper placed on the above-mentioned media supplemented with 200 μM AS and incubated for 3 days in the dark at 25 ± 1°C. The control treatment consisted of noninfected calluses without sonication. Transformation with other bacterial strains was performed using the optimized parameters, and the efficiency was documented.

### Selection, regeneration, and hardening of transgenic plants

The cocultivated somatic embryogenic masses were washed three times with timentin (300 mg l^−1^) solution, blot-dried, and cultured on SHS1 containing 30 mg l^−1^ hygromycin/25 mg l^−1^ kanamycin and 300 mg l^−1^ timentin (SHS1T300K25/H30) for selection of transgenic plants. The embryogenic masses were subcultured at 2–3-week intervals on a medium containing 150 mg l^−1^ timentin and 30 mg l^−1^ hygromycin/50 mg l^−1^ kanamycin (SHS1T150K50/H30). Subsequently, 10 independent somatic embryo clusters were transferred individually into SH liquid (50 ml) medium with 3% glucose and 100 mg l^−1^ (SHG3T100) timentin in 250 ml Erlenmeyer flasks. Different somatic embryo clusters were selected as a single somatic embryo cluster appeared to have been formed from a single transformed somatic embryo. Well-grown somatic embryo-derived plantlets were transferred to a soil mix of sand and potting soil (1:1) in small pots (4 × 4 cm), as previously described. The pH of all plant tissue culture media was adjusted to 5.8, and the media were sterilized in an autoclave at 15 lb pressure (121°C) for 15 min. Solid media were prepared by adding 0.8% (w/v) agar (PlantMedia, USA). Sterile media were poured into sterile Petri dishes of 20 (100 × 15 mm) and 40 ml (90 × 25 mm).

### Agroinfiltration of black pepper leaves

*A. tumefaciens* harboring binary plasmids containing *mgfp* were used for agroinfiltration. Bacterial cultures were grown overnight, as mentioned earlier. Bacterial cultures (25 ml) were prepared in half-strength MS liquid with 3% sucrose (w/v) and 200 μM AS and incubated for 1 h at 50 rpm on a rotary shaker. Following centrifugation, the pellet was dispersed in 25 ml of agroinfiltration buffer (10 mM N-morpholinoethanesulfonic acid, 10 mM MgCl_2_, pH 5.4) containing 200 μM AS and incubated at RT for 20–30 min (50 rpm). The bacterial suspension was carefully infiltrated into the adaxial surface of the fully expanded third and fourth leaves of six-week-old somatic embryo-derived plantlets grown in the plant room using a 1 ml syringe without the needle.

### Development of composite plants

*A. rhizogenes* ATCC15834 harboring the plasmid with *mgfp* was cultured in YM medium as described previously [61] until an OD_600_ of 0.5–0.7, and the pellet after centrifugation was resuspended in 2 ml half-strength MS medium (2% sucrose) with 200 μM AS. Somatic embryo-derived plantlets hardened in the greenhouse were carefully uprooted, and the primary roots were cut out at the root-shoot junction by a slant cut using a sterile scalpel. The cut ends of the plantlet stems and stem cuttings (from well-grown plants) were dipped in an agrobacterial culture for 30 min at RT. Infected shoot bases were blot dried. They were planted into a mix of sand and potting soil (1:1) in small pots (4 × 4 cm), covered with a moistened transparent plastic polyethylene bag, and kept in the plant room. The number of roots that showed GFP expression was documented after 30 days, and the plants were replanted in the soil mix.

### GUS, GFP, and RFP expression

Histochemical GUS assay of various tissues was conducted according to the protocol of Jefferson *et al*. [83]. Nontransgenic plants were used as controls. Plantlets and embryogenic masses transformed with the *gusA* construct were subjected to GUS assays as described by Sasi *et al*. [81]. The tissues were photographed using a D90 Digital camera (Nikon). Tissues were collected from at least three different lines of transgenic plants for further analysis. Three biological replicates of each tissue were used for GUS assays.

The expression of GFP and RFP/DsRED in transformed somatic embryos/tissues was observed and documented using a Leica Thunder Model Organism Microscope (Leica, Germany) with specific filters, as documented previously [61]. The transient (after 4 days of infection) and stable (after 20 days) expression of GFP and RFP in somatic embryos and GFP in the roots of the composite plants was documented.

### Genotyping by PCR

Genomic DNA was extracted from the control, transformed plants, and roots of composite plants using the cetyltrimethylammonium bromide (CTAB) extraction protocol [61, 84] with 50–100 mg of tissue. The DNA was precipitated using absolute ethanol, washed twice with 70% (v/v) ethanol, air-dried, and dissolved in 50 μl of 10 mM Tris–HCl buffer (pH 8.0).

Transformed plants (10 independent lines) and the roots of composite plants were confirmed by PCR using gene-specific primers of *mgfp*, *gusA*, and *dsRed* (Table S1) depending on the vector used for transformation. PCR reactions were performed in a volume of 20 μl containing 1× reaction buffer, 100 ng of DNA template, 1.5 mM MgCl_2_, 0.2 mM of each of the dNTPs, 2.5 units of HotStar Taq DNA polymerase (HotStar Taq DNA polymerase kit, Qiagen, Hilden, Germany), and 0.2 μM gene-specific forward and reverse primers. *Mgfp* (753 bp), *gusA* (353 bp), *rfp* (753 bp), and *dsRed* (681 bp) were amplified using gene-specific primers for *mgfp, gusA*, and *dsRed,* respectively. Wild-type (nontransformed) plants served as negative controls, and the respective plasmid DNAs were used as positive controls. The reaction conditions were as follows: 95°C for 15 min, 25 cycles of 94°C for 20 s, 57°C for 20 s (48°C for *dsRed*), 72°C for 40 s, and 7 min extension at 72°C. The PCR reaction mixtures were analyzed in 1.2% (w/v) agarose gels in TAE containing 1× Hydragreen with 1 kb plus DNA ladder (Thermo Scientific, USA) and documented using Geldoc (EZ Imager, Bio-Rad).

### Western blotting

Protein extracts were prepared as described by Zubco and Day [85], with some modifications. Briefly, 200 mg of frozen tissues (untransformed and different transformed lines expressing GFP) were homogenized in 600 μl of buffer (25 mM Tris–HCl, pH 7.5, 10% (v/v) glycerol, 10% (w/v) SDS, 0.5% (v/v) β-mercaptoethanol), boiled for 3 min and centrifuged at 14000 rpm for 10 min. The collected supernatant was subjected to SDS-PAGE. SDS-PAGE was performed on a Gradient 4%–15% (Bio-Rad, USA) gel loading 20 μl of total protein. A PageRuler™ Prestained Protein Ladder, ranging from 10 to 180 kDa (Thermo Scientific™, Cat no. 26616), was used as a protein size standard. Western blot was carried out using the Genscript 1-h Western blot kit (cat. no. L00241, USA) according to the manufacturer’s protocol using a GFP antibody (cat. no. A-11122, Agrisera, USA). The blot was captured using a Li-Cor C-DiGit Western Blot Scanner (USA).

### Target sequence of *PnPds* and genome editing

The phytoene desaturase (*Pds*) sequence of black pepper was obtained from the whole-genome sequence (WGS) of black pepper [86]. The *Pds* sequence of *Piper colubrinum* (GenBank Acc no: KM582050.1) was aligned with the WGS of black pepper, and the region that showed >50% identity was identified. The conserved sequence in the aligned region (within the exon) was used to design a specific target for *PnPds* using CRISPRdirect (https://crispr.dbcls.jp/). The 20 base sequence was flanked by adaptors on both forward CPnPDS-F and reverse CPnPDS-R primers (Table S1) synthesized were assembled using the Goldengate protocol (NEB, USA) into pHSE401 (https://www.addgene.org/62202/) harboring kanamycin for bacterial and hygromycin for plant selection, and transformed into *E. coli* (DH5α) cells. To assess the potential off-target effects, an off-target analysis was performed using Cas-OFFinder (http://www.rgenome.net/cas-offinder/). Positive colonies were selected on LB plates with kanamycin by colony PCR. PCR reactions were performed in a volume of 20 μl containing 1× reaction buffer, 100 ng of DNA template, 1.5 mM MgCl_2_, 0.2 mM of each of the dNTPs, 2.5 units of HotStar Taq DNA polymerase (HotStar Taq DNA polymerase kit, Qiagen, Hilden, Germany), and 0.2 μM of the M13 forward primer and reverse primer (CPnPDS-R) of the target. The reaction conditions were as follows: 95°C for 15 min; 25 cycles of 94°C for 20 s, 55°C for 20 s, 72°C for 30 s, and 7 min extension at 72°C. The target duplex in the transformed plasmid was also confirmed by sequencing the extracted plasmid from the positive colonies using a plasmid extraction kit (Qiagen). The sequence-confirmed plasmid was electroporated into *A. tumefaciens* strain AGL1. Genome editing of *PnPds* was performed using the optimized transformation procedure described herein.

To confirm genome editing, PCR primers for *PnPds* were designed using Primers3 (https://primer3.ut.ee/). DNA was extracted from putative genome-edited plants (10 independent lines) as described previously, and PCR was performed using 100 ng of DNA with the designed forward and reverse PnPDSFL primers (Table S1). PCR was performed as described previously, and the amplicons were extracted using a gel extraction kit (Macherey-Nagel, USA). Editing of the *PnPds* gene was confirmed using Sanger sequencing (Macrogen, Korea).

### Temporary immersion bioreactor (TIB) system

Scale-up plant production was attempted using the TIB (RITA, Vitropic, France) system with an ACO-9610 air pump (Hailea, China). Early embryonic cultures of untransformed, GFP expressing and *Pds-*edited lines of Sreekara were grown in TIB. Initially, SHS1 medium (200 ml, antibiotic-free) was used to culture the embryogenic mass, and at the onset of cotyledonary embryo development (after 15 days), it was replaced with SHG3 (200 ml, antibiotic-free) for plantlet growth. The immersion times for embryogenic cultures were 5 and 10 min (12 h intervals, 7 a.m. and 7 p.m.). The light and temperature conditions for the TIB were the same as those for the suspension cultures. The plantlets produced through TIB were hardened as previously described.

### Gene expression analysis

Total RNA was extracted from different tissues as described by Sasi *et al*. [87]. RNA from somatic embryo samples (embryos, roots, and cotyledons separately) grown in the light collected from liquid SH medium with 1% and 3% sugars (sucrose, glucose, maltose, fructose, sorbitol, and a combination of glucose and fructose (0.5% or 1.5% each) were used to analyze the expression levels of different genes during embryo conversion to plantlets (Table S1). RNA extracted from the leaves, stems, and roots of wild-type (control), *gusA-*transformed PCR-positive, and *Pds-*edited plants of black pepper was used to evaluate their expression. Nanodrop2000 (ThermoScientific) was used to quantify the extracted RNA, and a 1% (w/v) agarose gel was used to assess the RNA quality. Following the manufacturer’s instructions, 1 μg of total RNA was converted into first-strand cDNA using the QuantiTect Reverse Transcription Kit (Qiagen, Germany). Quantitative real-time PCR (qRT-PCR) was performed using SYBR Green PCR master mix (Applied Biosystems, USA), diluted cDNA samples, and *gusA*/*PnPds* primers (Table S1) in an optical 96-well plate using a StepOnePlus™ Real-Time PCR System to determine target gene expression profiles. The qRT-PCR program was as follows: 10 min at 95°C, 40 cycles of 95°C for 3 s, and 30 s at 60°C. The transcript levels of the different genes were determined using the 2^−ΔΔ^Ct method [88]. *PnGAPDH* (*Arabidopsis* ortholog, AT1G79530.1) was used as the reference gene (Table S1). Three technical replicates were analyzed for each biological replicate.

### Statistical analysis

The transient and stable transformation frequencies are the means of three independent experiments ±SE. All transformation experiments and tests for antibiotic tolerance were performed in triplicate. Statistical analyses were performed using Microsoft Excel. Student’s *t*-test/Duncan Multiple Range Test (DMRT) was performed in relevant experiments to assess the level of significance (*P* < 0.05).

## Supplementary Material

Web_Material_uhag067

## Data Availability

All data supporting the findings of this study are included in this manuscript.
