# Supplementary material for: Reducing recalcitrance of black pepper to Agrobacterium-mediated transformation: an efficient way through nucellar apomixis to establish transgenic and genome-edited plants at high frequency and scale-up through bioreactor
Source: Hortic Res. 2026 Feb 28;13(6):uhag067. doi: 10.1093/hr/uhag067 (PMC13253342; doi:10.1093/hr/uhag067)
Supplement: Web_Material_uhag067 [file web_material_uhag067.zip › Suppl Tables .docx]

Table S1: Primers used in the study

| Primer | Sequence (5’ – 3’) |
| --- | --- |
| mGFP-F | TGTCAGTGGAGAGGGTGAAGG |
| mGFP-R | ACAGGGCCATCGCCAATTGGA |
| DsRED-F | ATGGCGCGCTCCTCCAAGAACG |
| DsRED-R | CTACAGGAACAGGTGGTGGCGG |
| GUS-F | ATGAACATGGCATCGTGGTGATTG; |
| GUS-R | GAGATCGCTGATGGTATCGGTGTG |
| GUS-qF | GAATACGGCGTGGATACGTTAG |
| GUS-qR | GATCAAAGACGCGGTGATACA |
| PnGAPDH-qF | ATGAAGGATTGGCGAGGTGG |
| PnGAPDH-qR | AGGCCATTCCAGTGAGCTTC |
| CPnPDS-F | ATTGACGGTTTGACAGTCAAAGAA |
| CPnPDS-R | AAACTTCTTTGACTGTCAAACCGT |
| PnPDSFL-F | GTGGAAGGAACACTCTATGATC |
| PnPDSFL-R | GGGTAGGAGAACAGCAACAA |
| PnPDS-qF | TGACAGTCAAAGAATGGATGC |
| PnPDS-qR | ATGCCAATTATTGAGTACCACA |
| PnSUS2-qF | CAACATCCTCGCCACCTT |
| PnSUS2-qR | CTGAAGCCTAAGAGAAGCAAGA |
| PnPIN2-qF | ATACCAGCCATTTGCAATGAAG |
| PnPIN2-qR | AGCCATGGAGAAAGAAGCATAG |
| PnOSM-qF | ACCGTGTTTAAGACCGACCA |
| PnOSM-qR | ACCATTTCATGGGCAAAAGA |
| PnDHN-qF | AGCAGATCAGCTGGAAGGAA |
| PnDHN-qR | ATCAGTGGCACATTGTTCA |

Table S2 Fold difference in the expression of genes in cotyledons and roots and their ratio with respect to the sugars (3%)

| Genes | Difference in fold expression of cotyledon and root | | | Fold expression ratio of cotyledon: root ratio | | |
| --- | --- | --- | --- | --- | --- | --- |
|  | Sucrose | Glucose | Fructose | Sucrose | Glucose | Fructose |
| *PIN2* | 0.6 | 0.3 | 0.1 | 1.42 | 0.6 | 3.94 |
| *Osm* | -1.02 | -2.38 | 1.81 | 0.5 | 0.19 | 1.83 |
| *Sus2* | 0.0004 | -0.0043 | -0.0037 | 1.36 | 0.2 | 0.37 |
| *Dhn1* | 0.0036 | -0.0021 | -0.0071 | 0.669 | 0.074 | 0.294 |
