# Supplementary figures and images for: Reducing recalcitrance of black pepper to Agrobacterium-mediated transformation: an efficient way through nucellar apomixis to establish transgenic and genome-edited plants at high frequency and scale-up through bioreactor
Source: Hortic Res. 2026 Feb 28;13(6):uhag067. doi: 10.1093/hr/uhag067 (PMC13253342; doi:10.1093/hr/uhag067)

## Slide 1
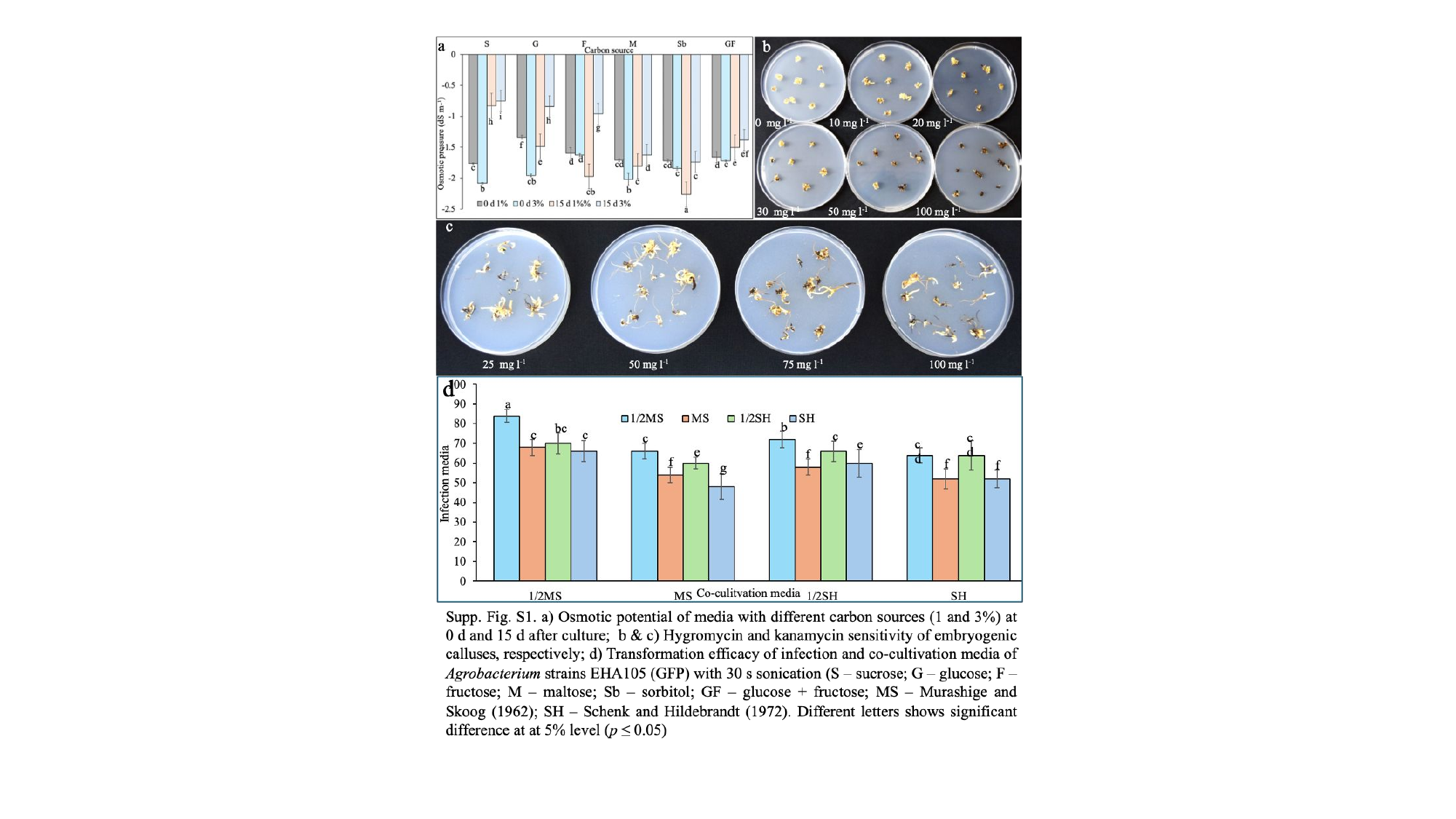

## Slide 2
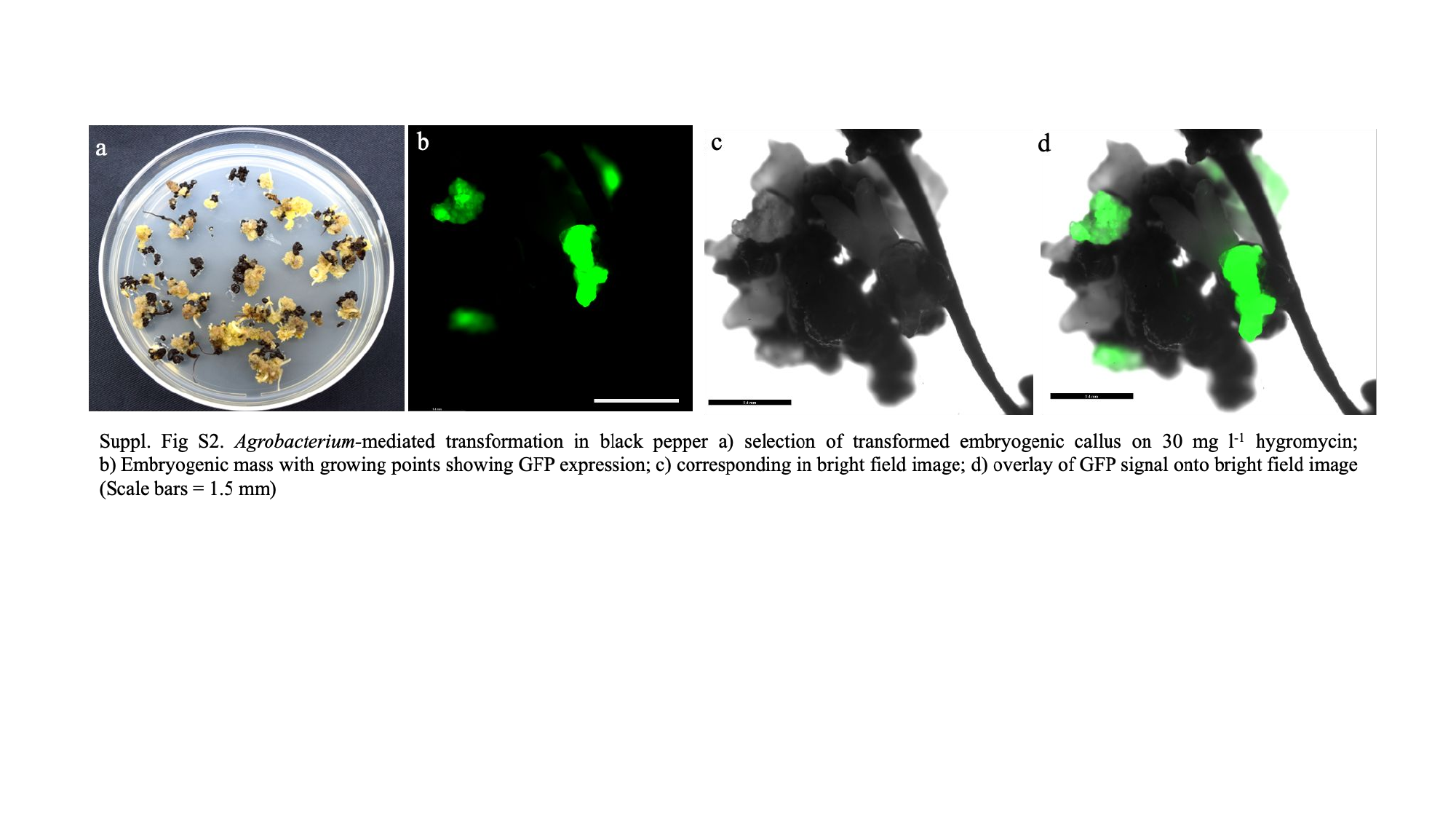

## Slide 3
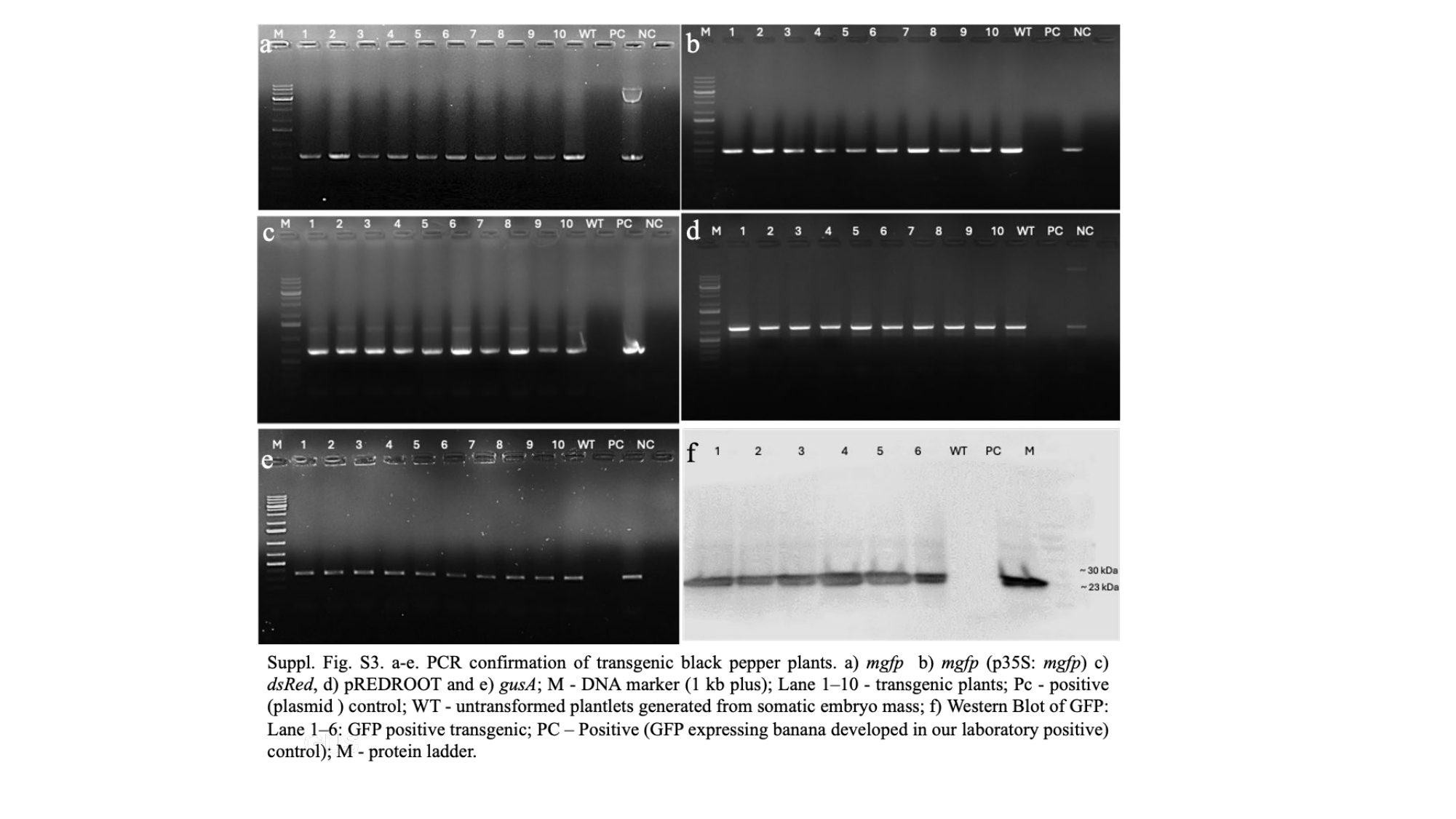

Supplement: Web_Material_uhag067 [file web_material_uhag067.zip › Suppl Figures .pptx]
